# Radioimmunotherapy of pancreatic cancer xenografts in nude mice using ^90^Y-labeled anti-α_6_β_4_ integrin antibody

**DOI:** 10.18632/oncotarget.9631

**Published:** 2016-05-26

**Authors:** Winn Aung, Atsushi B. Tsuji, Hitomi Sudo, Aya Sugyo, Yoshinori Ukai, Katsushi Kouda, Yoshikazu Kurosawa, Takako Furukawa, Tsuneo Saga

**Affiliations:** ^1^ Diagnostic Imaging Program, Molecular Imaging Center, National Institute of Radiological Sciences, Chiba, Japan; ^2^ Perseus Proteomics Inc., Tokyo, Japan; ^3^ Innovation Center for Advanced Medicine, Fujita Health University, Toyoake, Japan

**Keywords:** targeted radioimmunotherapy, pancreatic tumor, α_6_β_4_ integrin, yttrium-90, radiolabeled antibody

## Abstract

The contribution of integrin α_6_β_4_ (α_6_β_4_) overexpression to the pancreatic cancer invasion and metastasis has been previously shown. We have reported immunotargeting of α_6_β_4_ for radionuclide-based and near-infrared fluorescence imaging in a pancreatic cancer model. In this study, we prepared yttrium-90 labeled anti-α_6_β_4_ antibody (^90^Y-ITGA6B4) and evaluated its radioimmunotherapeutic efficacy against pancreatic cancer xenografts in nude mice. Mice bearing xenograft tumors were randomly divided into 5 groups: (1) single administration of ^90^Y-ITGA6B4 (3.7MBq), (2) double administrations of ^90^Y-ITGA6B4 with once-weekly schedule (3.7MBq × 2), (3) single administration of unlabeled ITGA6B4, (4) double administrations of unlabeled ITGA6B4 with once-weekly schedule and (5) the untreated control. Biweekly tumor volume measurements and immunohistochemical analyses of tumors at 2 days post-administration were performed to monitor the response to treatments. To assess the toxicity, body weight was measured biweekly. Additionally, at 27 days post-administration, blood samples were collected through cardiac puncture, and hematological parameters, hepatic and renal functions were analyzed. Both ^90^Y-ITGA6B4 treatment groups showed reduction in tumor volumes (*P* < 0.04), decreased cell proliferation marker Ki-67-positive cells and increased DNA damage marker p-H2AX-positive cells, compared with the other groups. Mice treated with double administrations of ^90^Y-ITGA6B4, exhibited myelosuppression. There were no significant differences in hepatic and renal functions between the 2 treatment groups and the other groups. Our results suggest that ^90^Y-ITGA6B4 is a promising radioimmunotherapeutic agent against α_6_β_4_ overexpressing tumors. In the future studies, dose adjustment for fractionated RIT should be considered carefully in order to get the optimal effect while avoiding myelotoxicity.

## INTRODUCTION

Pancreatic cancer is a malignant disease with poor prognosis. According to the Globocan 2012 data, the estimated numbers of new cases and deaths caused by pancreatic cancer worldwide in 2012 were about 338000 and 331000, respectively [1]. Pancreatic cancer treatment options are limited to surgery, systemic chemotherapy, and radiation therapy, and the outcomes are not yet fully satisfactory. New therapeutic strategies and diagnostic imaging modalities are needed. There is a renewed interest in the targeted irradiation by radioimmunotherapy (RIT), using radiolabeled monoclonal antibodies, in order to achieve target-specific accumulation of lethal doses in pancreatic tumor while avoiding the toxic effects on the adjacent normal tissue.

Integrins are a family of transmembrane glycoproteins composed of various α and β subunit heterodimers, which are responsible for cell-cell and cell-extracellular matrix interactions [2]. They are involved in cancer progression, including adhesion, migration, invasion, proliferation, survival, and metastasis [3]. Tumor-associated functions of integrin α_6_β_4_ (α_6_β_4_) are starting to be recognized. Integrin α_6_β_4_ was initially identified as an epithelial-specific integrin [4]. Afterward, it was identified as an antigen associated with metastasis [5], and the significant overexpression of α_6_β_4_ was observed in some carcinomas in comparison with normal tissue. It has been proposed that the function of α_6_β_4_ is substantially altered as normal epithelia undergo malignant transformation and progress to the invasive carcinoma, and that this integrin contributes to the behavior of aggressive carcinoma cells [6–12]. The overexpression of α_6_β_4_ in pancreatic adenocarcinoma has been demonstrated by immunohistochemical (IHC) analyses [13, 14] and gene profiling studies [15]. Previously, we have isolated a fully human monoclonal IgG_1_ antibody against α_6_β_4_, ITGA6B4, from a large-scale human antibody library constructed using a phage-display system and screened using living pancreatic cancer cells [16]. According to ELISA results, our ITGA6B4 antibody was strongly reacts with human α_6_β_4_ antigen but its cross-reactivity to murine α_6_β_4_ antigen is negligible (Supplementary Figure 1). We labeled it with indium-111 (^111^In) for single-photon emission computed tomography (SPECT) and with the near-infrared (NIR) fluorophore indocyanine green (ICG) for NIR fluorescence imaging [17]. SPECT and NIR imaging demonstrated that α_6_β_4_-expressing tumors exhibited good uptake of these probes. Here, in accordance with the theranostic concept, we labeled ITGA6B4 with yttrium-90 (^90^Y). ^90^Y is a pure β-emitter with a high energy level (maximum energy, 2283 keV), long range maximum tissue penetration (11 mm) and an appropriate half-life (64.1 h), which makes it a suitable radionuclide for RIT [18]. It is also a residualizing radiometal that is retained in the cell upon internalization [19].

RIT is a promising method for cancer treatments. RIT with dose fractionation is expected to provide better therapeutic outcomes, allowing larger amounts of radionuclides to be administered, by reducing the heterogeneity of the absorbed doses and diminishing hematologic toxicity [20]. In this study, we tested single (3.7MBq) and double administrations with once-weekly schedule of ^90^Y-ITGA6B4 (3.7MBq × 2) in mice bearing pancreatic cancer xenografts. The treatment efficacy was evaluated, and the toxicity of each regimen was assessed and reported here.

## RESULTS

### RIT effects on the tumor growth in nude mice

**Figure 1:**
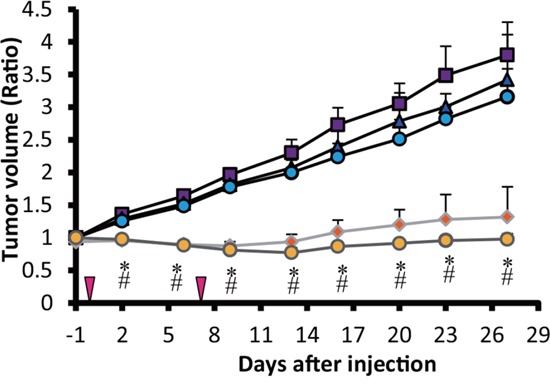
Radioimmunotherapy effects on BxPC-3 xenografts Tumor volume change expressed as the ratio of the volume at the indicated day/volume 1 day before the start of the treatment. Tumors in mice treated with a single administration (3.7MBq) (orange diamonds) and double administration (3.7MBq × 2) (yellow circles) of ^90^Y-ITGA6B4, showed significantly reduced growth rates compared with the untreated control (purple squares), unlabeled single dose of ITGA6B4 treated (blue triangles) and double administration of unlabeled ITGA6B4 treated (light blue circles) groups. Values shown represent mean ± SD, ^*^P < 0.04 (single ^90^Y-ITGA6B4 *vs* others), ^#^*P* < 0.04 (double ^90^Y-ITGA6B4 *vs* others), n=7, except in a group that received double administration of ^90^Y-ITGA6B4, numbering 6 mice from the 22^nd^ day onward. Vertical arrowheads indicate the day of administration.

Tumors in mice that received single (3.7MBq) and double administrations of ^90^Y-ITGA6B4 (3.7MBq × 2) showed growth delay, compared with the other groups: untreated control group, unlabeled ITGA6B4 single administration group and unlabeled ITGA6B4 double administration group. Significant differences in tumor volume between groups were observed from day 2 onward. However, there was no significant difference between single and double administration of ^90^Y-ITGA6B4 groups (Figure 1). Estimated radiation absorbed doses for ^90^Y-ITGA6B4 by tumor and tissues are summarized in Table 1, and the resulting tumor-to-normal tissue dose ratios (TNTDR) are also included.

**Table 1:** Radiation absorbed doses estimated from the biodistribution of radiolabeled ITGA6B4 in BxPC3-xenografted mice

Tissues	^90^Y-ITGA6B4		
	Gy/MBq	Gy/3.7MBq	TNDTR^a^
**Lung**	3.1	11.3	2.7
**Liver**	2.0	7.5	4.0
**Spleen**	2.0	7.4	4.1
**Pancreas**	0.6	2.1	14.3
**Stomach**	0.6	2.3	13.1
**Intestine**	0.7	2.7	11.2
**Kidney**	2.2	8.0	3.8
**Muscle**	0.3	1.2	25
**Bone**	0.8	3.0	10
**Tumor (BxPC-3)**	8.1	30.1	

a TNTDR = Tumor-to-normal tissue dose ratio

Time point (1.5, 24, 48, and 96 h after administration)

### Evaluation of the RIT effects using immunohistochemical analysis

**Figure 2:**
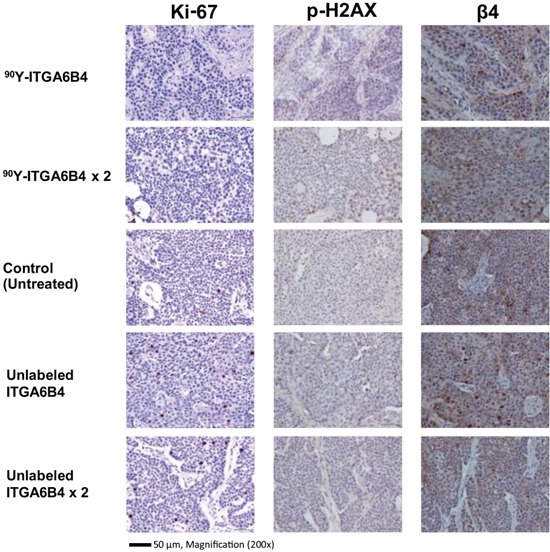
Ki-67, p-H2AX immunostaining of tumor sections 2 days after radioimmunotherapy A reduction in Ki-67-positive cell numbers and an increased p-H2AX-positive cell numbers were observed in mouse samples treated with single (3.7MBq) or double administrations (3.7MBq × 2) of ^90^Y-ITGA6B4, compared with untreated and matched control samples. Similar integrin β_4_ expression pattern was seen in all tumor samples. Tumor section photos were taken under 200× magnification (scale bar, 50 μm).

Cell proliferation marker Ki-67-positive cell numbers were noticeably decreased in the tumors of mice treated with single or double administrations of ^90^Y-ITGA6B4, compared with the other groups. In contrast, DNA damage marker phospho-Histone H2AX (p-H2AX)-positive cell numbers were increased in the tumors of mice treated with single or double administrations of ^90^Y-ITGA6B4, compared with the other groups. On the other hand, TUNEL-positive cell were rare and there was no clear difference in the TUNEL-staining patterns between the groups (Data not shown). Immunohistochemical staining with β_4_ antibody depicted the almost similar expression pattern in all tumors (Figure 2).

### The assessment of RIT toxicity

**Figure 3:**
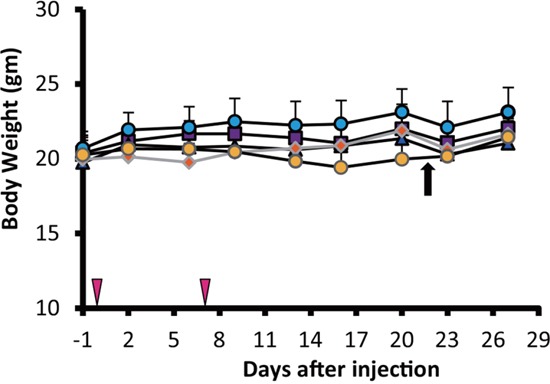
Comparison of the body weights of tumor-bearing nude mice The average mouse body weight did not differ significantly among all 5 groups. One mouse in the double administration of ^90^Y-ITGA6B4 (3.7MBq × 2) group died after 22 days (indicated with black arrow). ^90^Y-ITGA6B4 single (3.7MBq) (orange diamond) and double administration (3.7MBq × 2) (yellow circles), untreated control (purple squares), unlabeled ITGA6B4 single administration (blue triangles) and unlabeled ITGA6B4 double administration (light blue circles) groups are presented. Values shown represent mean ± SD, n=7, except in the double administration of ^90^Y-ITGA6B4 group, which numbered 6 mice from the 22^nd^ day onward. Vertical arrowheads indicate the day of administration.

**Figure 4:**
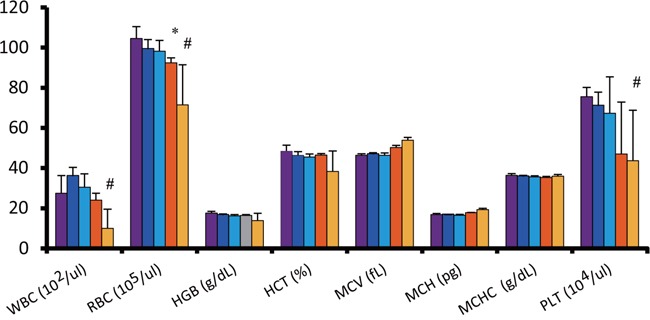
Radiation-induced hematologic toxicity Hematological parameters were analyzed 27 days after radioimmunotherapy. Myelosuppression was observed, evident from the decreased RBC, WBC, and platelet counts, in mice treated with double administrations of ^90^Y-ITGA6B4 (3.7 MBq × 2), while a decreased RBC count was noticed in mice that received single administration (3.7 MBq). Values shown represent mean ± SD, ^*^P < 0.05 (single ^90^Y-ITGA6B4 *vs* untreated), ^#^*P* < 0.05 (double ^90^Y-ITGA6B4 *vs* untreated). Untreated control (purple, n=4), single administration (orange, n=3), double administrations (yellow, n=4) of ^90^Y-ITGA6B4, unlabeled ITGA6B4 single administration (blue, n=3), and unlabeled ITGA6B4 double administrations (light blue, n=4).

**Figure 5:**
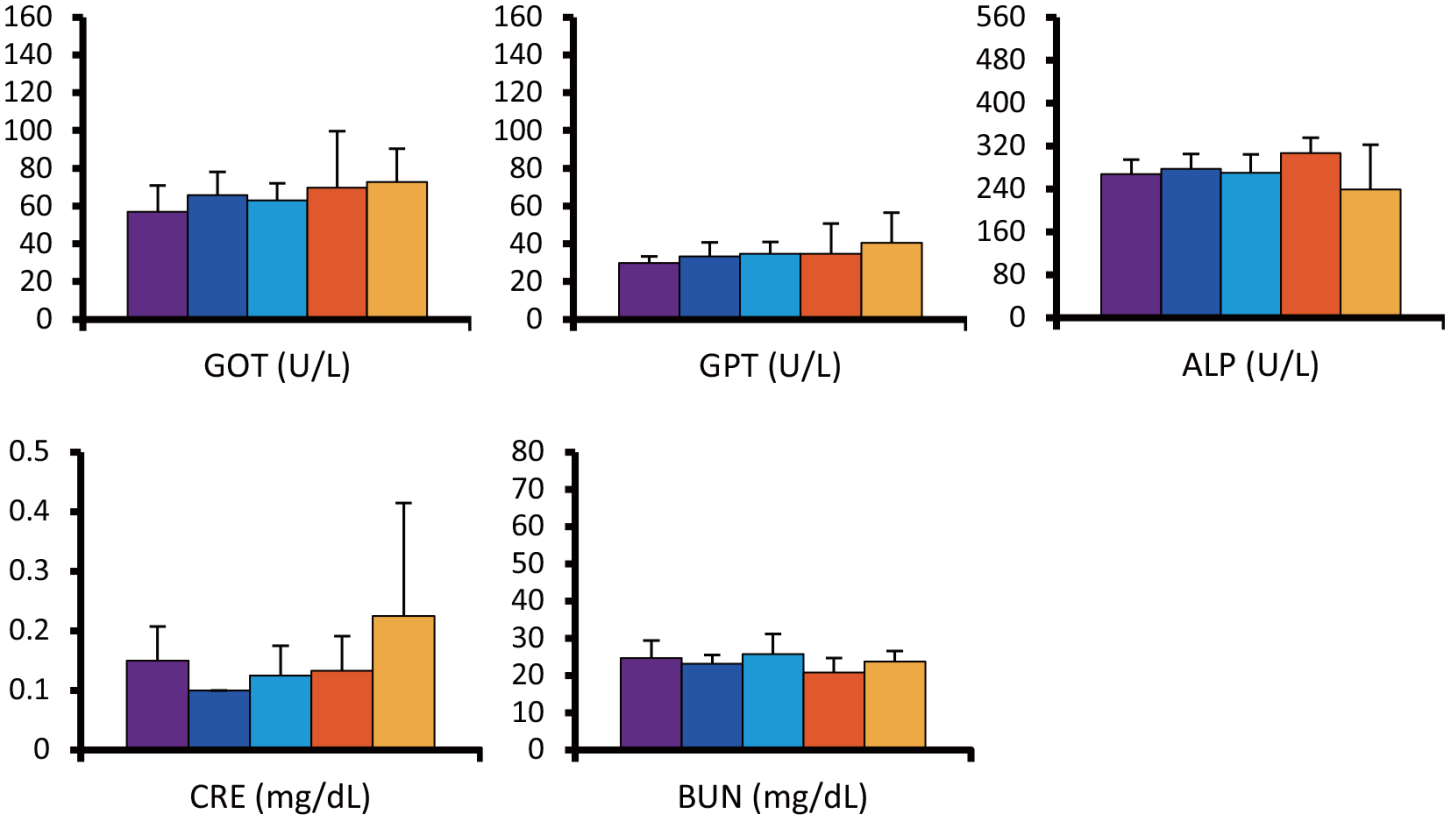
Radiation-induced hepatic and renal toxicity No abnormally increased values of serum GOT, GPT, ALP values for liver function, and serum CRE and BUN for kidney function, were observed. Values shown represent the mean ± SD. Untreated control (purple, n=4), single administration (3.7MBq) (orange, n=3), double administrations (3.7MBq × 2) (yellow, n=4) of ^90^Y-ITGA6B4, unlabeled ITGA6B4 single administration (blue, n=3), and unlabeled ITGA6B4 double administrations (light blue, n=4).

Although mice injected with radiolabeled antibody showed slight decrease in average body weight post-administration, no significant differences were observed between the groups throughout the experiment (Figure 3). However, one mouse from the double administration of ^90^Y-ITGA6B4 group developed pale skin and few petechiae during the study, and it died at 22 days post-treatment. The tested hematological parameters at 27 days after the start of therapy showed that the mice treated with the double administrations of ^90^Y-ITGA6B4 had significantly decreased red blood cell [RBC; mean (71.5 ± 19.9) × 10^5^/μl (68.4% of the untreated control)], white blood cell [WBC; mean (10.0 ± 9.5) × 10^2^/μl (36.4% of the untreated control)] and platelet counts [mean (43.7 ± 25.2) × 10^4^/μl (57.8% of the untreated control)] (Figure 4). In the mice treated with the single administration of the same ^90^Y-ITGA6B4 dose, only the RBC count was significantly decreased [mean (92.5 ± 2.4) × 10^5^/μl (88.5% of the untreated control)]. Additional biochemical tests showed minimal side effect of RIT on liver and renal functions. The values of serum GOT (glutamic oxaloacetic transaminase), GPT (glutamic pyruvate transaminase), ALP (alkaline phosphatase) values and serum CRE (creatine) and BUN (blood urea nitrogen) were not increased (Figure 5). Though serum GOT values in all groups were slightly over the reference limit, there was no significant differences between these groups, and we considered the liver toxicity as non-existent.

## DISCUSSION

Integrin α_6_β_4_ facilitates multiple aspects of malignant progression, such as proliferative signaling, tumor invasion and metastasis, evasion of apoptosis, and stimulation of angiogenesis. Additionally, overexpression of α_6_β_4_ in different types of cancer was associated with poor prognosis [21]. Previously, we proposed α_6_β_4_ as a potential target for pancreatic cancer imaging and showed the specific accumulation of ITGA6B4 in α_6_β_4_ overexpressing tumor [17]. Here, we labeled anti-α_6_β_4_ antibody with β-emitting radionuclide ^90^Y and reconfirmed, using PET imaging, that this antibody (^90^Y-ITGA6B4) accumulates in tumor. Although the software and hardware improvements are necessary, the ^90^Y-ITGA6B4 PET images were successfully acquired, validating the localization of the antibody in target tumor, through the CT image co-registration (Supplementary Figure 2). The main purpose of current study was to observe the radioimmunotherapeutic effects of ^90^Y-ITGA6B4 on the pancreatic cancer xenografts in mice. We chose the ^90^Y, because it has higher energy and longer particle range, allowing to more radioactivity in the tumor cell and to a more uniform irradiation in large tumors that may have heterogeneous uptake [22]. Although an alternative therapeutic β-emitter, Lutenium-177, that has lower energy and a shorter range of tissue penetration, has been reported in the treatment of small tumors [23], our present study was performed only with ^90^Y. The xenografts we used were derived from the human pancreatic α_6_β_4_-overexpressing cancer cell line BxPC-3.

We carried out the tumor volume and immunohistochemical analyses, for the comprehensive evaluation of the ^90^Y-ITGA6B4 radiotherapeutic efficacy, and demonstrated that it has significant antitumor effects. We used longitudinal monitoring of tumor progression and regression in a single animal throughout the course of treatment, and found that both single and double administrations of ^90^Y-ITGA6B4 significantly delayed tumor growth in comparison with the control groups. We expected that double administrations will be more potent than single administration, but no significant difference was observed (Figure 1). We examined the potential effects of RIT on proliferation, DNA double-stranded breaks and apoptosis of cancer cells by immunohistochemical staining, which can detect Ki-67-, p-H2AX-, TUNEL-positive cells. Ki-67, a nuclear protein, is proliferation marker [24], and p-H2AX is DNA damage marker [25]. We found that the number of Ki-67-positive cells was reduced and the number of p-H2AX-positive cells was increased in the tumors of mice that received RIT, in comparison with the other groups (Figure 2). Apoptotic cells were rare in the TUNEL-stained sections, and the staining patterns in all groups were almost identical (Data not shown). The apoptosis was evaluated at 2 days post-administration, and a further analysis of serial post-administration time points may lead to different findings. These results are in line with the results of our previous RIT study using ^90^Y-anti-transferrin receptor antibody (^90^Y-TSP-A01) [26]. Furthermore, we observed previously that BxPC-3 tumors show decreased radiosensitivity and abundant cancer-associated stroma, and most of the blood vessels in these tumors are surrounded by the stromal tissue [26] which could lead to the reduced penetration of macromolecules, such as IgG [27]. In comparison with ^111^In-TSP-A01 uptake in BxPC-3 tumor [26], the uptake of our new antibody, ^111^In-ITGA6B4, in same tumor was superior [17]. *In vivo* activity of ^90^Y-ITGA6B4 may be predicted from a biodistribution surrogate, ^111^In-ITGA6B4, because ^111^In- and ^90^Y-labeled antibodies, proteins, and peptides are biologically similar [28]. We have previously investigated the biodistribution of ^111^In- ITGA6B4 in mice bearing pancreatic cancer tumor xenografts. The tumor-absorbed dose up to 4 days after injection for ^90^Y-ITGA6B4 (3.7 MBq), estimated from the acquired biodistribution data, was 30.1 Gy, while this dose was 17.9 Gy for ^90^Y-TSP-A01 [26]. Both tumor uptake and tumor-absorbed doses suggest that ^90^Y-ITGA6B4 accumulation is favorable for the effective radiotherapy. We have investigated only BxPC-3 xenograft tumors, and this may be a limiting factor, but the anticancer effects of ^90^Y-ITGA6B4 in solid tumors are probably more cytostatic than cytocidal. Investigations using different tumors, with different α_6_β_4_ expression levels, tumor vasculature, and vasculature permeability are needed in order to confirm this.

^18^F-FDG-PET is widely applied in clinical and often in some preclinical settings, in order to evaluate radio- and chemotherapy responses [29–33], and to predict the therapeutic outcomes [34]. Although using the change of FDG uptake to assess the radiation response in RIT is not well established, we measured tumor FDG uptake (SUV_max_) as a trial, in order to assess the therapeutic efficacy of RIT. At 1 day before the RIT, the baseline tumor FDG uptake in all groups was almost the same. At 6 and 13 days after the initial dose of RIT, significantly reduced FDG uptake was seen in the both single and double administrations of ^90^Y-ITGA6B4 treated groups compared with the other three groups. No significant difference in the tumor uptake was observed between 2 groups that received single and double administrations of ^90^Y-ITGA6B4 (Supplementary Figure 3). SUV_max_ decreased to about 70% of the baseline value during the first week but no further decrease was observed at day 13. Although we found the decreased FDG uptake in the BxPC-3 xenografts following ^90^Y-ITGA6B4 administration, detailed observation of factors determining the ^18^F-FDG uptake: such as glucose transporters expression, activities of hexokinase and glucose-6-phosphate of tumors were needed to be checked. An alternative PET radiotracer 3′-^18^F-fluoro-3′-deoxy-L-thymidine (FLT), a surrogate biomarker of tumor proliferation, is attractive choice for the treatment response assessment. Cell proliferation, as immunohistochemical analyses showed, was particularly inhibited by ^90^Y-ITGA6B4 treatments, and therefore, incorporating FLT results in our study would even further confirm our results, but unfortunately, we were unable to use it.

Animal body weights and general conditions throughout the treatment were monitored, and laboratory tests were performed to evaluate the toxicity of ^90^Y-ITGA6B4. We found that the single administrations of ^90^Y-ITGA6B4 (3.7 MBq) did not induce a pronounced myelosuppression, liver toxicity, renal toxicity, or body weight loss. On the other hand, anemia, leucopenia and thrombocytopenia, which appear alongside a pronounced myelosuppression, developed in mice treated with the double administrations of ^90^Y-ITGA6B4 (3.7 MBq × 2). No abnormal liver and kidney function were observed. One mouse from this group developed pale skin and petechiae, and was dead on the 22^nd^ day following RIT, and we suggest that the cause of death was myelotoxicity caused by an overdose. We could not estimate when myelosuppression started because the serial laboratory and blood tests were not performed. We learned that 3.7 MBq ^90^Y-ITGA6B4 is a tolerable, therapeutically efficient dose when delivered as a single injection, but the double administrations could be perilous. A fractionated administration, i.e., splitting a larger dose of more than 3.7 MBq into a number of smaller ones, in order to improve the therapeutic response, has not been tested and this study should be performed in the future. Fine-tuning of the number of administrations, time intervals between the administrations and radioactivity per administration, could lead to the determination of the best therapeutic regimen.

In conclusion, the current study reveals that a single dose of ^90^Y-ITGA6B4 (3.7 MBq) effectively inhibited tumor growth *in vivo*, in mice bearing human pancreatic cancer xenografts overexpressing α_6_β_4_, without producing any noticeable adverse effects, while double administrations of the same dose with once-weekly schedule caused myelotoxicity. Taken together, our results support a further development of α_6_β_4_ targeted pancreatic cancer RIT and provide the base for the rational dose-adjustment, in order to achieve the optimal fractionation schemes for RIT.

## MATERIALS AND METHODS

### Cells

Human pancreatic cancer cell line BxPC-3 was purchased from American Type Culture Collection (Manassas, VA, USA) and passaged in Roswell Park Memorial Institute (RPMI) 1640 medium (Sigma-Aldrich, St. Louis, MO, USA) supplemented with 10% fetal bovine serum (FBS, Nichirei Biosciences, Tokyo, Japan), 100 U/mL penicillin G sodium, and 100 mg/mL streptomycin sulfate (Invitrogen, Carlsbad, CA, USA) at 37°C in a humidified atmosphere containing 5% CO_2_.

### Subcutaneous pancreatic mouse xenograft tumor model

All animal experiments were performed in accordance with the animal experimentation protocol approved by the Animal Care and Use Committee of National Institute of Radiological Sciences. A suspension of 5 × 10^6^ BxPC-3 cells in 100 μL RPMI medium was mixed with BD matrigel matrix (BD Biosciences, Bedford, MA, USA) and subcutaneously inoculated into the right thigh of nude mice (7-week-old female BALB/cA Jcl-nu/nu mice, CLEA, Shizuoka, Japan).

### Antibody radiolabeling

Human anti-α_6_β_4_ monoclonal antibody (IgG_1_) was labeled with ^90^Y, as previously reported [35]. Briefly, the antibody solution and a chelating agent, *N*-[(*R*)-2-amino-3-(*p*-isothiocyanato-phenyl)propyl]-*trans*-(*S*,*S*)-cyclohexane-1,2-diamine-*N*,*N*,*N*′,*N*′,*N*′-pentaacetic acid (CHX-A′′-DTPA) (Macrocyclics, Dallas, TX, USA) were mixed in a molar ratio of 1:2.5 and incubated overnight at 37°C. The conjugation ratio of DTPA and antibody was estimated to be 1.6, calculated from the ratio of ^90^Y-DTPA-antibody to ^90^Y-DTPA, determined by isoelectric focusing. Unconjugated DTPA was removed using a Sephadex G-50 column (GE Healthcare, Little Chalfont, UK). Afterward, DTPA-conjugated antibody (83 μg in 0.1 M sodium acetate buffer [pH 6.0]) was incubated with the mixture of ^90^Y-chloride (74 MBq, Eckert & Ziegler Radiopharma GmbH, Berlin, Germany) and 1 M sodium acetate buffer (pH 6.0) for 30 min at room temperature (RT). The radiolabeled antibody was purified using a Sephadex G-50 column (730× *g* for 2 min). The radiochemical purity determined by TLC was > 99%. The radiochemical yield was approximately 85 to 90%, and the specific activity was approximately 758 to 802 kBq/μg.

### Radioimmunotherapy (RIT) with ^90^Y-ITGA6B4

When the subcutaneous tumors reached approximately 8-10 mm in the longest diameter, at 5 weeks after inoculation, mice bearing xenograft tumors were randomly divided into 5 groups (n=7 for each group). Group (1) received a single administration of ^90^Y-ITGA6B4 (3.7MBq), group (2) received double administrations of ^90^Y-ITGA6B4 with once-weekly schedule (3.7MBq × 2), group (3) received a single administration of unlabeled ITGA6B4, group (4) received double administrations of unlabeled ITGA6B4 with the once-weekly schedule, and group (5) received no injections. Each quantity of the injected antibody was adjusted to 20 μg, by the addition of the intact antibody. Tumor- and tissue-absorbed doses for ^90^Y-labeled antibody were estimated from the biodistribution data of ^111^In-labeled antibody [26]. ^90^Y emits β-particles with a mean emitted energy of 0.9331 MeV, therefore the mean energy emitted per transition was calculated as 1.496 × 10^−13^ Gy kg (Bq s)^−1^, since 1 eV is equal to 1.602 × 10^−19^ J [36]. The calculation procedure of absorbed dose from the biodistribution data was previously described for other radiolabeled antibodies [37]. Briefly, tumor uptake at various time points was plotted against time, and the area under the curve (AUC) was calculated. The doses absorbed up to 4 days after injection were estimated using AUC and mean energy emitted per transition as follows: AUC × 1.495 × 10^−13^ × injected activity.

### Evaluation of treatment efficacy by measuring tumor volumes

To monitor the tumor response, tumor volumes were measured twice a week throughout the experiment, using caliper, and approximated using the equation: volume (mm^3^)=(length [mm])×(width [mm])^2^/2. Relative tumor volume was calculated as the volume at the indicated day divided by the volume at 1 day before start of the treatment.

### Immunohistochemical tumor analysis

At 2 days after the treatment with ^90^Y-ITGA6B4 or unlabeled ITGA6B4, tumor samples from 1 mouse of each group (n=1) were extirpated, fixed in 4% paraformaldehyde, and embedded in paraffin. Untreated tumor was used as control. Ki-67 staining of 4 μm thick sections was performed using an anti-human Ki-67 polyclonal antibody (Dako Denmark, Glostrup, Denmark), as previously described [38]. Phospho-Histone H2AX (p-H2AX) staining was also detected using anti-human p-H2AX (Ser 139) (20E3) monoclonal antibody (Cell Signaling technology, Danvers, MA, USA). To depict the β_4_ expression in tumor cells, rabbit anti-human β_4_ integrin (H-101) polyclonal antibody (Santa Cruz Biotechnology, CA, USA) was used as primary antibody. HRP-linked polymer anti-rabbit (Dako Denmark), was used as secondary antibody. To detect the apoptotic tumor cells, terminal deoxynucleotidyl transferase-mediated deoxyuridine triphosphate nick end labeling (TUNEL) staining with an ApopTag Peroxidase In Stiu Apoptosis Detection Kits (Millipore Corporation, Temecula, CA, USA) was performed. Each slide was observed by using microscope Olympus BX43 system (Olympus, Tokyo, Japan).

### ^18^F-FDG PET Imaging

FDG-PET scans were conducted before, and at 6 and 13 days after antibody administration. Tumor bearing mice from each group (n=3-4) were intravenously injected with ^18^F-FDG (2.0 MBq). At 50 min following the injection, static PET data acquisition was conducted for 10 min, using a small-animal PET system (Inveon, Siemens Medical Solutions, Malvern, PA), under 1.5% isoflurane anesthesia. Images were reconstructed using a maximum a posteriori (MAP) method with attenuation correction using Inveon Acquisition Workplace software (Siemens Medical Solutions). Using ASIPro VM software (CTI Concorde Microsystems, Knoxville, TN, USA), regions of interest (ROIs) were manually drawn on 3 slices in coronal, transverse, and sagittal planes of each tumor, and the standardized uptake value (SUV_max_) was measured in each ROI, for the quantitative analysis.

### Treatment toxicity assessment

To assess the toxicity, body weight of each mouse was measured every 3-4 days. At 27 days after the first administration, all mice were euthanized. Their blood samples were collected through cardiac puncture, and the hematological parameters and biochemical parameters relevant to hepatic and renal functions were analyzed. We used an automated hematology analyzer, Celltac Alpha (Nihon Kohden, Tokyo, Japan), for hematological and automated clinical chemistry analyzer, DRI-CHEM 7000V (Fujifilm, Tokyo, Japan), for biochemical tests.

### Statistical analysis

Significant differences between the groups were determined by Student's *t*-test (Excel, Microsoft, Redmond, WA, USA). *P*-values < 0.05 were considered significant.

## SUPPLEMENTARY FIGURES



## References

[1] Ferlay J, Soerjomataram I, Dikshit R, Eser S, Mathers C, Rebelo M, Parkin DM, Forman D, Bray F (2015). Cancer incidence and mortality worldwide: sources, methods and major patterns in GLOBOCAN 2012. Int J Cancer.

[2] Hynes RO (1992). Integrins: versatility, modulation, and signaling in cell adhesion. Cell.

[3] Desgrosellier JS, Cheresh DA (2010). Integrins in cancer: biological implications and therapeutic opportunities. Nat Rev Cancer.

[4] Sonnenberg A, Calafat J, Janssen H, Daams H, Vanderraaijhelmer LMH, Falcioni R, Kennel SJ, Aplin JD, Baker J, Loizidou M, Garrod D (1991). Integrin-Alpha-6-Beta-4 complex is located in hemidesmosomes, suggesting a major role in epidermal-cell basement-membrane Adhesion. J of Cell Biol.

[5] Falcioni R, Sacchi A, Resau J, Kennel SJ (1988). Monoclonal antibody to human carcinoma-associated protein complex: quantitation in normal and tumor tissue. Cancer Res.

[6] Rabinovitz I, Mercurio AM (1996). The integrin alpha 6 beta 4 and the biology of carcinoma. Biochem Cell Biol.

[7] Mercurio AM, Rabinovitz I (2001). Towards a mechanistic understanding of tumor invasion-lessons from the alpha6beta 4 integrin. Semin Cancer Biol.

[8] Bon G, Folgiero V, Di Carlo S, Sacchi A, Falcioni R (2007). Involvement of alpha(6)beta(4) integrin in the mechanisms that regulate breast cancer progression. Breast Cancer Research.

[9] Soung YH, Gil HJ, Clifford JL, Chung J (2011). Role of alpha6beta4 integrin in cell motility, invasion and metastasis of mammary tumors. Curr Protein Pept Sci.

[10] Beaulieu JF (2010). Integrin alpha6beta4 in colorectal cancer. World J Gastrointest Pathophysiol.

[11] Noh TW, Soung YH, Kim HI, Gil HJ, Kim JM, Lee EJ, Chung J (2010). Effect of {beta}4 integrin knockdown by RNA interference in anaplastic thyroid carcinoma. Anticancer Res.

[12] Lipscomb EA, Mercurio AM (2005). Mobilization and activation of a signaling competent alpha6beta4integrin underlies its contribution to carcinoma progression. Cancer Metastasis Rev.

[13] Cruz-Monserrate Z, Qiu S, Evers BM, O'Connor KL (2007). Upregulation and redistribution of integrin alpha6beta4 expression occurs at an early stage in pancreatic adenocarcinoma progression. Mod Pathol.

[14] Gleason B, Adley B, Rao MS, Diaz LK (2005). Immunohistochemical detection of the beta4 integrin subunit in pancreatic adenocarcinoma. J Histochem Cytochem.

[15] Logsdon CD, Simeone DM, Binkley C, Arumugam T, Greenson JK, Giordano TJ, Misek DE, Kuick R, Hanash S (2003). Molecular profiling of pancreatic adenocarcinoma and chronic pancreatitis identifies multiple genes differentially regulated in pancreatic cancer. Cancer Res.

[16] Kurosawa G, Akahori Y, Morita M, Sumitomo M, Sato N, Muramatsu C, Eguchi K, Matsuda K, Takasaki A, Tanaka M, Iba Y, Hamada-Tsutsumi S, Ukai Y (2008). Comprehensive screening for antigens overexpressed on carcinomas via isolation of human mAbs that may be therapeutic. Proc Natl Acad Sci U S A.

[17] Aung W, Tsuji AB, Sudo H, Sugyo A, Furukawa T, Ukai Y, Kurosawa Y, Saga T (2016). Immunotargeting of Integrin alpha6beta4 for Single-Photon Emission Computed Tomography and Near-Infrared Fluorescence Imaging in a Pancreatic Cancer Model. Mol Imaging.

[18] Kassis AI (2008). Therapeutic radionuclides: biophysical and radiobiologic principles. Semin Nucl Med.

[19] Sharkey RM, Behr TM, Mattes MJ, Stein R, Griffiths GL, Shih LB, Hansen HJ, Blumenthal RD, Dunn RM, Juweid ME, Goldenberg DM (1997). Advantage of residualizing radiolabels for an internalizing antibody against the B-cell lymphoma antigen, CD22. Cancer Immunol Immunother.

[20] DeNardo GL, Schlom J, Buchsbaum DJ, Meredith RF, O'Donoghue JA, Sgouros G, Humm JL, DeNardo SJ (2002). Rationales, evidence, and design considerations for fractionated radioimmunotherapy. Cancer.

[21] Stewart RL, O'Connor KL (2015). Clinical significance of the integrin alpha6beta4 in human malignancies. Lab Invest.

[22] de Jong M, Breeman WA, Valkema R, Bernard BF, Krenning EP (2005). Combination radionuclide therapy using 177Lu- and 90Y-labeled somatostatin analogs. J Nucl Med.

[23] Brouwers AH, van Eerd JE, Frielink C, Oosterwijk E, Oyen WJ, Corstens FH, Boerman OC (2004). Optimization of radioimmunotherapy of renal cell carcinoma: labeling of monoclonal antibody cG250 with 131I, 90Y, 177Lu, or 186Re. J Nucl Med.

[24] Scholzen T, Gerdes J (2000). The Ki-67 protein: from the known and the unknown. J Cell Physiol.

[25] Rogakou EP, Pilch DR, Orr AH, Ivanova VS, Bonner WM (1998). DNA double-stranded breaks induce histone H2AX phosphorylation on serine 139. J Biol Chem.

[26] Sugyo A, Tsuji AB, Sudo H, Okada M, Koizumi M, Satoh H, Kurosawa G, Kurosawa Y, Saga T (2015). Evaluation of efficacy of radioimmunotherapy with 90Y-labeled fully human anti-transferrin receptor monoclonal antibody in pancreatic cancer mouse models. PLoS One.

[27] Minchinton AI, Tannock IF (2006). Drug penetration in solid tumours. Nat Rev Cancer.

[28] Onthank DC, Liu S, Silva PJ, Barrett JA, Harris TD, Robinson SP, Edwards DS (2004). 90Y and 111In complexes of a DOTA-conjugated integrin alpha v beta 3 receptor antagonist: different but biologically equivalent. Bioconjug Chem.

[29] Contractor KB, Aboagye EO (2009). Monitoring predominantly cytostatic treatment response with 18F-FDG PET. J Nucl Med.

[30] Avril NE, Weber WA (2005). Monitoring response to treatment in patients utilizing PET. Radiol Clin North Am.

[31] Veeravagu A, Liu Z, Niu G, Chen K, Jia B, Cai W, Jin C, Hsu AR, Connolly AJ, Tse V, Wang F, Chen X (2008). Integrin alphavbeta3-targeted radioimmunotherapy of glioblastoma multiforme. Clin Cancer Res.

[32] Niu G, Sun X, Cao Q, Courter D, Koong A, Le QT, Gambhir SS, Chen X (2010). Cetuximab-based immunotherapy and radioimmunotherapy of head and neck squamous cell carcinoma. Clin Cancer Res.

[33] Jeraj R, Bradshaw TJ, Simoncic U (2015). Molecular Imaging to Plan Radiotherapy and Evaluate its Efficacy. J Nucl Med.

[34] Czernin J, Phelps ME (2002). Positron emission tomography scanning: current and future applications. Annu Rev Med.

[35] Sogawa C, Tsuji AB, Sudo H, Sugyo A, Yoshida C, Odaka K, Uehara T, Arano Y, Koizumi M, Saga T (2010). C-kit-targeted imaging of gastrointestinal stromal tumor using radiolabeled anti-c-kit monoclonal antibody in a mouse tumor model. Nucl Med Biol.

[36] Wong FC (2009). MIRD: Radionuclide Data and Decay Schemes. J Nucl Med.

[37] Yoshida C, Tsuji AB, Sudo H, Sugyo A, Kikuchi T, Koizumi M, Arano Y, Saga T (2013). Therapeutic efficacy of c-kit-targeted radioimmunotherapy using 90Y-labeled anti-c-kit antibodies in a mouse model of small cell lung cancer. PLoS One.

[38] Sudo H, Tsuji AB, Sugyo A, Ogawa Y, Sagara M, Saga T (2012). ZDHHC8 knockdown enhances radiosensitivity and suppresses tumor growth in a mesothelioma mouse model. Cancer Sci.

