# Supplementary material for: Radioimmunotherapy of pancreatic cancer xenografts in nude mice using 90Y-labeled anti-α6β4 integrin antibody
Source: Oncotarget. 2016 May 26;7(25):38835–44. doi: 10.18632/oncotarget.9631 (PMC5122433; doi:10.18632/oncotarget.9631)
Supplement: Supplementary file 1 [file oncotarget-07-38835-s001.pdf]

Radioimmunotherapy of pancreatic cancer xenografts in nude mice using ^{90}Y-labeled anti-$\alpha_6\beta_4$ integrin antibody

SUPPLEMENTARY FIGURES

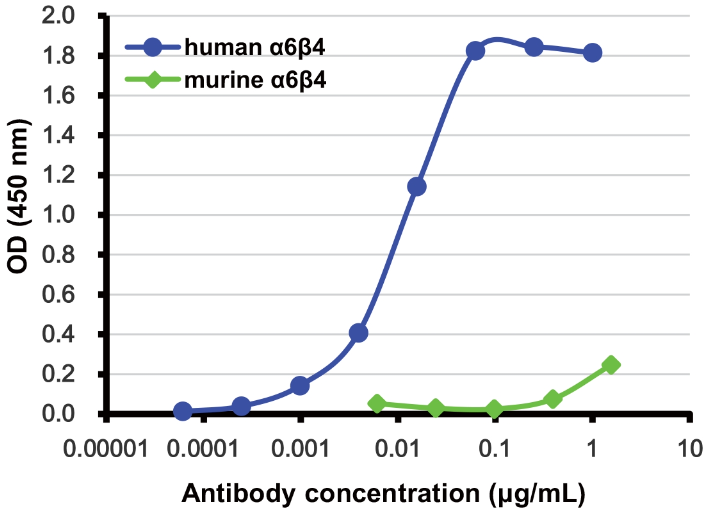

Supplementary Figure S1: Binding of ITGA6B4 antibody to human $\alpha_6\beta_4$ antigen and murine $\alpha_6\beta_4$ antigen. In an ELISA experiment, a dose titration of ITGA6B4 was added to immobilized human $\alpha_6\beta_4$ antigen or murine $\alpha_6\beta_4$ antigen. ITGA6B4 bound to human antigen with a higher affinity (Blue) but its cross-reactivity to murine antigen was negligible (Green).

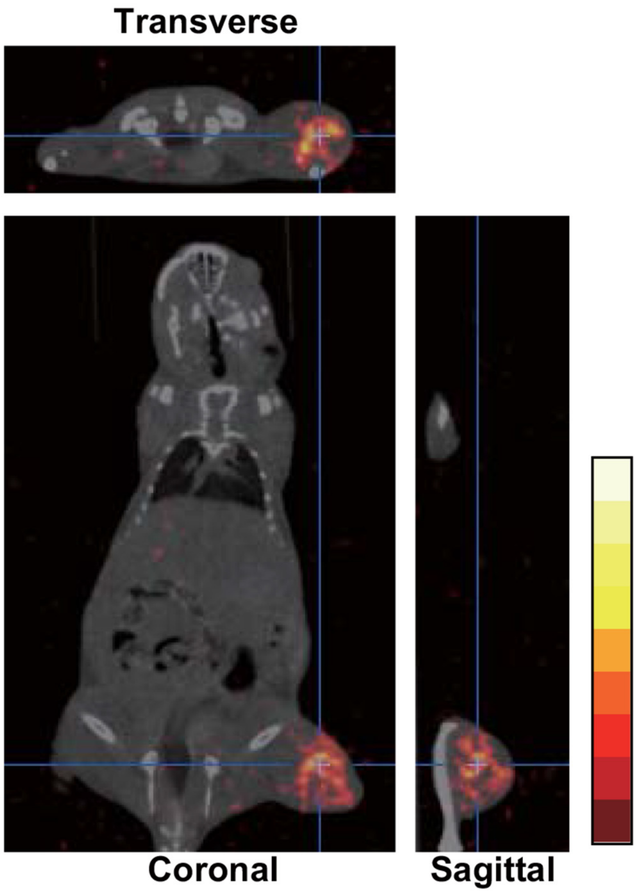

Supplementary Figure S2: Representative ^{90}Y-ITGA6B4 PET/CT images (coronal, transverse and sagittal) of a tumor xenograft mouse. The intersections of two blue lines indicate the corresponding subcutaneous tumor. Two days after intravenous injection of 7.4 MBq of ^{90}Y-ITGA6B4, static PET data acquisition was conducted for 120 min. The reconstructed image was obtained by setting the positron peak at the 411 keV-611 keV. Mouse fixed in the same bed position was transferred to the *in vivo* micro x-ray computed tomographic (CT) system R_mCT2 (Rigaku Co., Tokyo, Japan) scanner immediately after PET scanning. The whole-body image was taken with an exposure time of 2 min, tube voltage of 90 kV, 200 mA current, and an FOV of 60 mm in diameter. Images were acquired with the image reconstruction, visualization, and analysis program provided by the manufacturer. Both PET and CT data sets were coregistered to confirm the anatomic location of tumors. Fused images were obtained using PMOD PET data analysis software (PMOD Technologies, Zurich, Switzerland).

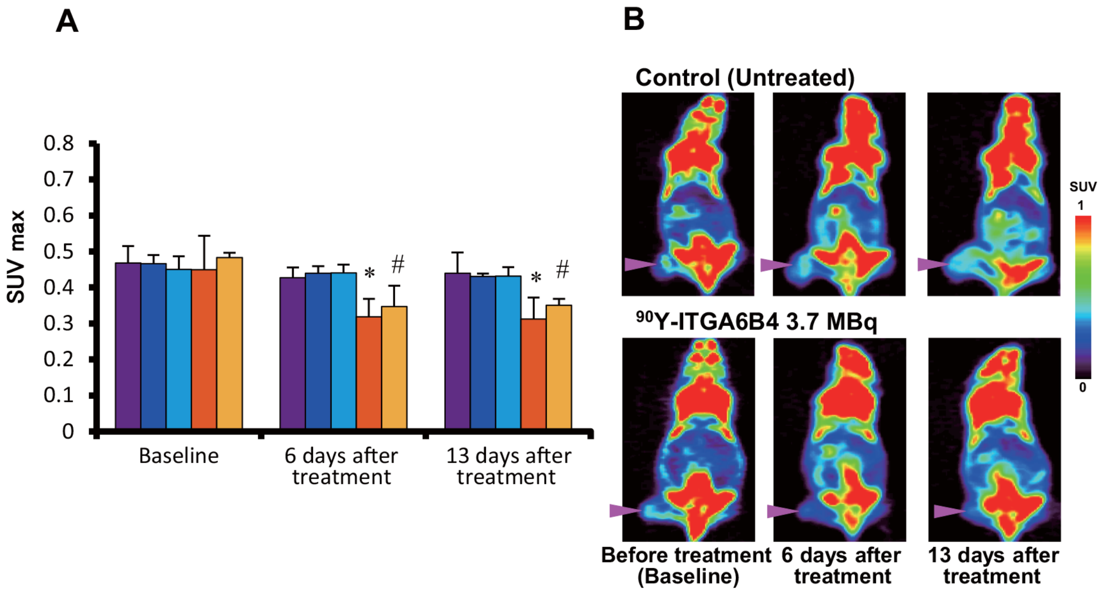

Supplementary Figure S3: Tumor uptake of ^{18}F-FDG. A. At 6 and 13 days after the first dose of RIT, the uptake (SUV_{max}) in the tumors of mice that received both single (3.7MBq) (orange, n=4) and double administrations (3.7MBq x 2) (yellow, n=4) of ^{90}Y-ITGA6B4 significantly decreased, in comparison with the untreated control (purple, n=4), unlabeled ITGA6B4 single administration (blue, n=3) and unlabeled ITGA6B4 double administration (light blue, n=3) groups. Values shown represent mean ± SD, $^{*}P < 0.05$ (single ^{90}Y-ITGA6B4 *vs* others), $^{\#}P < 0.05$ (double ^{90}Y-ITGA6B4 *vs* others). **B.** The representative serial coronal PET images of 2 mice from the untreated control and ^{90}Y-ITGA6B4 single administration groups are shown. Arrowheads indicate subcutaneous tumors.
